# Using event-related brain potentials to evaluate motor-auditory latencies in virtual reality

**DOI:** 10.3389/fnrgo.2023.1196507

**Published:** 2023-07-05

**Authors:** Sascha Feder, Jochen Miksch, Sabine Grimm, Josef F. Krems, Alexandra Bendixen

**Affiliations:** ^1^Cognitive Systems Lab, Institute of Physics, Faculty of Natural Sciences, Chemnitz University of Technology, Chemnitz, Germany; ^2^Physics of Cognition Group, Institute of Physics, Faculty of Natural Sciences, Chemnitz University of Technology, Chemnitz, Germany; ^3^Research Group Cognitive and Engineering Psychology, Institute of Psychology, Faculty of Behavioural and Social Sciences, Chemnitz University of Technology, Chemnitz, Germany

**Keywords:** virtual reality (VR), electroencephalography (EEG), N1, P2, prediction, delay

## Abstract

Actions in the real world have immediate sensory consequences. Mimicking these in digital environments is within reach, but technical constraints usually impose a certain latency (delay) between user actions and system responses. It is important to assess the impact of this latency on the users, ideally with measurement techniques that do not interfere with their digital experience. One such unobtrusive technique is electroencephalography (EEG), which can capture the users' brain activity associated with motor responses and sensory events by extracting event-related potentials (ERPs) from the continuous EEG recording. Here we exploit the fact that the amplitude of sensory ERP components (specifically, N1 and P2) reflects the degree to which the sensory event was perceived as an expected consequence of an own action (self-generation effect). Participants (*N* = 24) elicit auditory events in a virtual-reality (VR) setting by entering codes on virtual keypads to open doors. In a within-participant design, the delay between user input and sound presentation is manipulated across blocks. Occasionally, the virtual keypad is operated by a simulated robot instead, yielding a control condition with externally generated sounds. Results show that N1 (but not P2) amplitude is reduced for self-generated relative to externally generated sounds, and P2 (but not N1) amplitude is modulated by delay of sound presentation in a graded manner. This dissociation between N1 and P2 effects maps back to basic research on self-generation of sounds. We suggest P2 amplitude as a candidate read-out to assess the quality and immersiveness of digital environments with respect to system latency.

## 1. Introduction

Since the beginning of digital technologies, determining suitable values for system latency has been a topic of scientific and practical interest (for a review, see Attig et al., 2017). *System latency* refers to the time interval between the user's input (e.g., pressing a button) and the system's response (e.g., producing a sound or updating the screen). Ideally, the system's response should follow the user's input at close-to-zero latency to mimic real-world physical interactions, yet this is impossible to achieve due to hardware and software constraints (e.g., Stauffert et al., 2016). Thus, instead of instantaneous system responses, digital technologies strive for responses at *acceptable* latencies. In determining what is acceptable, two aspects of system latency need to be distinguished: latency *delay* refers to the average time interval between user input and system response, while latency *jitter* refers to trial-to-trial variation in the time interval between user input and system response. Here we focus on latency *delay* and ask whether we can exploit the users' brain activity (measured via electroencephalography, EEG) to differentiate between latency delays in a virtual reality (VR) environment, using brain activity to system events not caused by user input as a reference.

There is consensus that strongly delayed system responses render the digital experience less natural, reduce the feeling of *presence* (i.e., “being there,” Skarbez et al., 2018) in the virtual environment, impede user performance as well as satisfaction with and acceptance of the system, and even cause motion sickness in some users (e.g., Stoner et al., 2011). At the same time, even in real-world physical systems humans are exposed to small delays (e.g., in the case of sound, as a consequence of travel time) and can learn to tolerate uncommon (yet reasonable) amounts of temporal mismatch between action and sensory consequence (e.g., Elijah et al., 2016; van Dam and Stephens, 2018). This adaptive capability might alleviate the need for extremely small latency delays. Exploiting this while avoiding the negative consequences of strongly delayed system responses requires an understanding of feasible boundaries of latency delay. Latency guidelines usually recommend specific limits for acceptable system delay; however, these recommendations are inconsistent across different guidelines (Attig et al., 2017). Inconsistencies are partly due to the methods chosen for evaluating the suitability of delay values, and partly due to actual differences in suitable values depending on the virtual environment and the nature of the interaction.

The adequacy of system latencies can be probed with different methods. First, participants can be asked to directly judge the system latency (e.g., Seow, 2008). This user-judgment approach has some downsides, the most prominent one being that asking the question frequently (“Do you have the impression that the system responds immediately to your actions?”) interferes with the process being measured—it reminds the user of the separation between the physical and virtual environments. It might even put the user into a “test mode” (Liebold et al., 2017) in which they devote attention to aspects of the virtual environment that they would not notice otherwise, leading to biased estimates of the actual effects of system latency. To avoid these complications, a second possibility is to measure the adequateness of system latency based on observable user behavior (i.e., without asking users). One can take measures of user performance (e.g., time and accuracy of completing certain tasks) and study whether task performance suffers from poor system latencies (e.g., Martens et al., 2018). This performance-based approach complements the judgment-based approach, with performance decrements even preceding the users' awareness of poor system latencies in some cases (Martens et al., 2018). Yet the user-performance approach requires precise knowledge of what the user is trying to achieve in order to interpret performance data in a meaningful way.

These challenges have led to a quest for psychophysiological methods that allow an unobtrusive on-line evaluation of the user state, including their currently experienced (in)adequacy of system latency. One approach is the measurement of *breaks in presence* (Slater and Steed, 2000): If the user experiences a sudden interruption in their interaction with the virtual environment, such as caused by a system glitch in terms of latency, this is reflected in physiological responses (Liebold et al., 2017) similar to a classical orienting response (OR; Sokolov, 1975). The promise of this approach is to turn the logic around for an unobtrusive on-line evaluation: Whenever an OR-like physiological signature is observed, system properties (including latencies) must be inspected for their adequacy. A challenge with this approach is that OR-like transient changes are ambiguous: Instead of breaks in presence, they could be due to more unspecific variation in user state, or they could constitute physiological responses to actual events in the virtual environment. To avoid these ambiguities, it is desirable to develop an approach that maintains the unobtrusive psychophysiological on-line evaluation while increasing the specificity of the inference (Wang and Suh, 2021).

In order to evaluate system latency more directly by psychophysiological methods, here we propose to use brain activity measured via EEG, and to capitalize on prior knowledge about EEG changes caused by sensory and motor events. From a continuous EEG recording, event-related potentials (ERPs) can be extracted, reflecting the brain's response to a particular event (e.g., a motor act or a sensory stimulus). From basic EEG and ERP research, it is well known that sensory ERPs are influenced not only by the physical properties of the eliciting sensory event, but also by the “inner state” of the recipient. Besides attentional aspects, a key factor is the predictability of the event (Baldeweg, 2006; Bendixen et al., 2009; Friston, 2010). All other things being equal, predictable sensory events elicit lower ERP responses than unpredictable events (see reviews by Bendixen et al., 2012; Denham and Winkler, 2017). Among the most predictable sensory events are those that were generated by the recipient him- or herself: We are not surprised by the sensory consequences of our own actions (Blakemore et al., 2000). The brain achieves this by activating a template of the predicted stimulus before any information reaches our sensory organs (SanMiguel et al., 2013a). If, however, the sensory consequences of the action do not match our expectations due to a production error (Sitek et al., 2013) or due to an external manipulation of the sensory stimulus (Heinks-Maldonado et al., 2005), the sensory ERP response increases back up to the level of an externally generated (thereby unpredictable) event. With appropriate control conditions, the amplitude of the sensory ERP response (more specifically, of the N1 and P2 components, see SanMiguel et al., 2013b) can thus be taken to reflect the degree to which the sensory event was perceived as an expected consequence of an own action. This so-called *N1/P2 suppression* or *self-generation effect* is a well-established finding in cognitive neuroscience, notwithstanding ongoing discussion of the precise mechanisms underlying it (e.g., review by Horváth, 2015).

Here we transfer the self-generation effect to the context of a virtual environment in order to assess ERP correlates of system latency. We hypothesize that sensory events in the VR that are generated by user actions show a characteristic reduction in ERPs relative to the same events being externally generated (i.e., a replication of the self-generation effect) as long as the events follow the user action with short latency delay. We further hypothesize that with considerable latency delay, users no longer perceive the sensory events as reliably resulting from their actions, and thus the ERPs resemble those of externally generated events (akin to Blakemore et al., 1999; for perceptual judgments). If these hypotheses can be confirmed, the ERPs could in turn be used as a readout of the suitability of system latency. Because the assessment rests on a comparison of ERPs elicited by specific self- vs. externally generated events precisely locked in time, the method is robust against unspecific variation in user state. Indeed, prediction-related ERPs have already been suggested as a quality metric for VR interactions (Gehrke et al., 2019, 2022). These authors introduced temporal mismatches between different sensory channels—specifically, between visual and haptic stimuli elicited by a user action. They show that such mismatches elicit a prediction-error-related ERP component, and illustrate how this could in turn be used for detecting visuo-haptic conflicts in VR (see also Alsuradi et al., 2021). Here we extend their approach for multisensory mismatches toward motor-sensory delays.

To test whether prediction-related ERPs reflect system latencies, a virtual environment is needed in which it is plausible for users to frequently elicit sensory stimuli by their actions, and in which the time delay of these stimuli is controllable in a highly precise manner. While the latter is a technical requirement that can be solved by (custom-built) hardware solutions, the former is a conceptual requirement that calls for VR construction in which transient sensory events can be naturally embedded. Transient sensory events with clear onsets facilitate the observation of the expected sequence of ERP components (e.g., P50-N1-P2 for sounds; see Alain and Winkler, 2012). Auditory events (as long as they are audible) lend themselves readily toward such an approach as it is guaranteed that the physical onset of the event will initiate physiological processing, independent of the users' current gaze direction or focus of attention. Indeed, many previous studies in basic lab settings (i.e., without the VR context) have used the auditory domain to study ERP correlates of sensorimotor prediction. In these studies, participants are provided with a response button to be pressed at certain time points. The button press elicits a sound whose processing is then compared against sounds appearing with no preceding button press (i.e., the externally-generated control condition). Several studies have shown that prolonging the interval between button press and sound onset (i.e., introducing delay) reduces the amount of N1/P2 suppression relative to an immediate presentation of the sound (Whitford et al., 2011; Elijah et al., 2016; Oestreich et al., 2016; Pinheiro et al., 2019). Pinheiro et al. (2019) argue for a temporal integration window in sensorimotor processing of about 200 ms, beyond which events would be perceived as externally generated. Yet some other studies have shown that even strongly delayed (by 500 to 1,000 ms) sounds can still lead to suppression of the sensory ERP components (Bäß et al., 2008). These conflicting results might relate to the reduced experimental setting in which participants actively wait for the sound to occur (since their task is to produce it; Bäß et al., 2008). Indeed, it is notoriously difficult to control the level of attention participants devote to the sounds in such tasks, and thus it has been argued that some observed differences might be confounded by attention (Saupe et al., 2013).

To move toward more naturalistic paradigms in which participants are not waiting for the sensory consequences of their actions, we propose to use motor acts that produce sounds more incidentally. Specifically, we have participants enter codes on virtual keypads to open doors in our VR. As with real keypads, entering a digit-based code is accompanied by sound. Yet the action's purpose and thus the participant's focus of attention is to open the door—not to generate the sound. The door-opening task makes use of the engaging nature of VR interactions and creates an enriched context compared to the typically reduced laboratory setting used to study sensorimotor prediction. The VR scenario also allows for plausible inclusion of the necessary control conditions: First, to study whether ERPs elicited by self-generated sounds are reduced in amplitude relative to ERPs elicited by externally generated sounds, we add a condition in which the virtual keypad eliciting the sounds is operated by a robot sphere, relieving participants from their task of opening the door occasionally. Second, for a fair comparison of ERPs elicited by self-generated and externally generated sounds, a motor-only control condition is needed to separate the motor and auditory parts of the self-generated sound ERP. This is realized by adding blocks in which operating the virtual keypad does not issue any sounds. Hence all conditions are embedded in the same VR scenario with an engaging task and *en passant* presentation of sounds and manipulation of their latency of occurrence.

In terms of latency delay between keypad operation and sound presentation, we contrast five different values: 10 ms (which was the lowest possible delay that could reliably be achieved with our custom-built hardware shortcutting the software-based VR latency delays), 50 ms and 100 ms (both of which are assumed to be tolerable by users based on typical guidelines; Attig et al., 2017), 150 ms (which is assumed to be tolerable by some guidelines but not others), and 300 ms (which is unanimously classified as too long; Attig et al., 2017). The chosen values also correspond with the ones used in non-VR-based settings manipulating latency delay (Whitford et al., 2011; Elijah et al., 2016; Oestreich et al., 2016; Pinheiro et al., 2019), thereby allowing for a direct comparison of the results.

We examine whether sensory ERPs elicited by self-generated sounds are modulated by latency delay in the described VR context, and how this relates to VR sounds not generated by the user. Finding a modulation by delay would add to the body of evidence regarding latency effects in sensorimotor prediction (Whitford et al., 2011; Elijah et al., 2016; Oestreich et al., 2016; Pinheiro et al., 2019), and would be promising in terms of using this effect for an unobtrusive evaluation of VR quality (akin to Gehrke et al., 2019, 2022).

## 2. Materials and methods

### 2.1. Participants

Twenty-four volunteers aged 19 to 35 from the Chemnitz University of Technology community participated in the study (20 women, 4 men; 22 right-handed, 2 left-handed; mean age: 23.9 years, SD: 3.5 years). Due to substantial artifacts in the EEG data, one participant's data were excluded from ERP data analysis (this participant was female and right-handed; mean age of the remaining sample: 24.0 years, SD: 3.6 years). All participants reported normal hearing and normal or corrected-to-normal vision. The study procedures were approved by the applicable local ethics review board (Ethikkommission HSW, Chemnitz University of Technology, case no. V-346-PHSFKS-Latenz-21072019). According to the Declaration of Helsinki, each participant gave written informed consent before the beginning of the experiment. This included full information about the background of the study, including the fact that the investigation aims at EEG correlates of latency differences. Participants were compensated for their participation at 10 EUR/h or with course credit.

### 2.2. Experimental stimuli and apparatus

#### 2.2.1. Virtual reality (VR) system with custom-built latency manipulation

**Figure 1:**
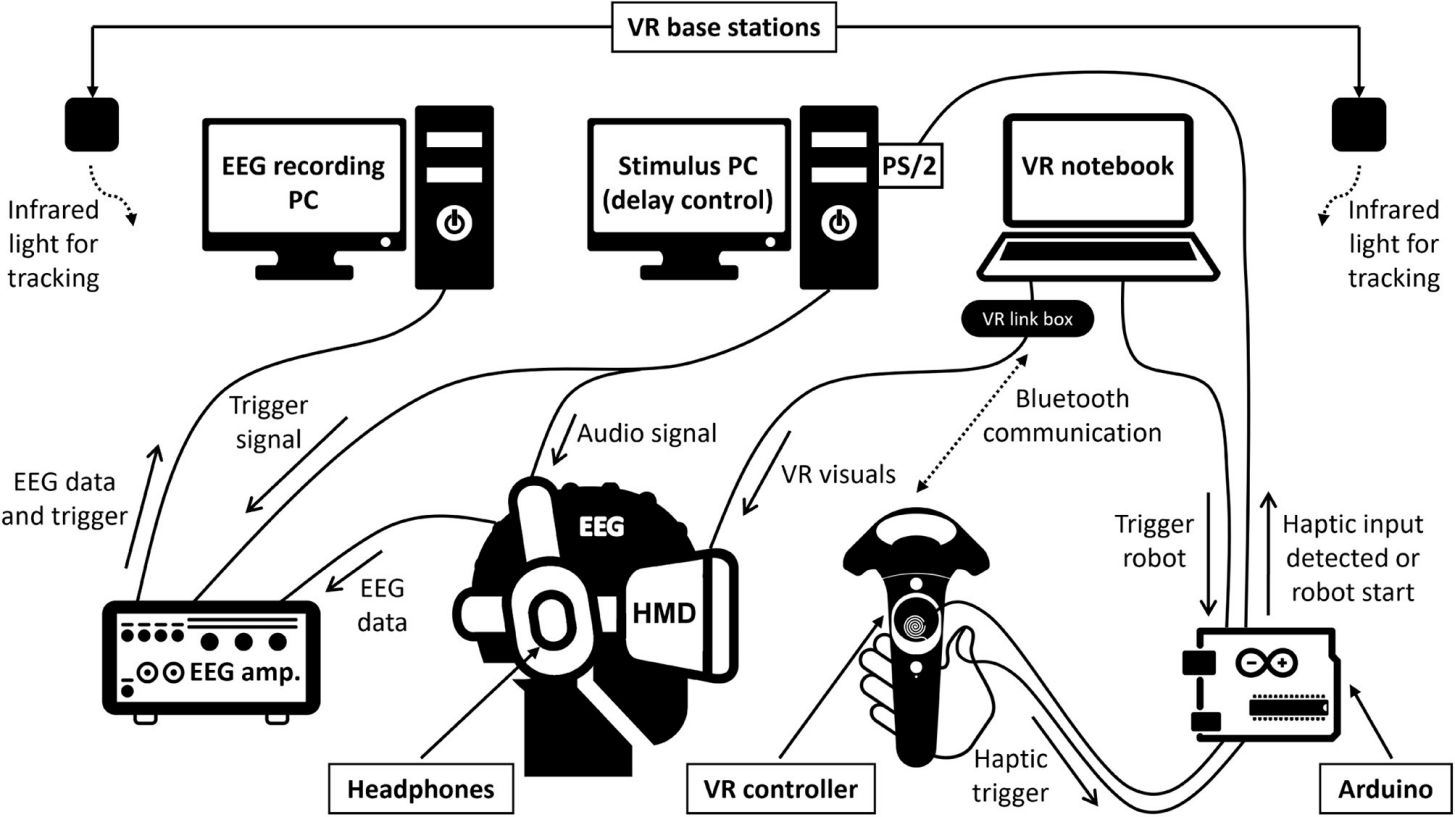
Schematic representation of the setup. The technical setup includes three synchronized computers and custom-built hardware elements with Arduino-based processing of touchpad responses detected by a copper wire coil to circumvent the VR controller's usual response latencies. Continuous lines indicate wired connections, dashed lines indicate wireless communication. See text for details.

**Figure 2:**
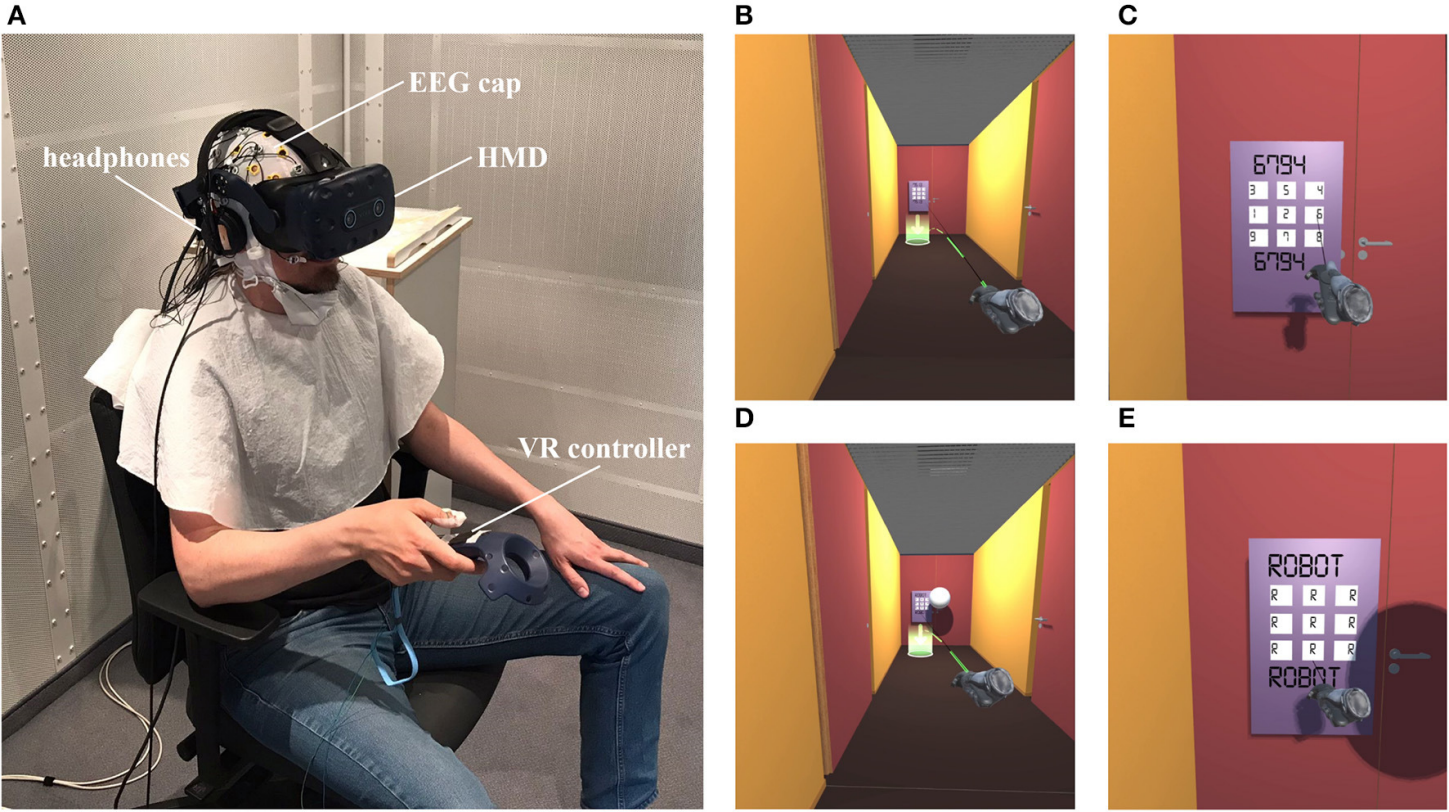
Snapshots of the VR setup and environment. **(A)** Head-mounted display (HMD), VR controller, headphones and EEG cap worn by the participant. The VR tracking devices and EEG amplifier are not visible on the photograph; all other setup components are placed outside of the experimental chamber. **(B)** View of the corridor from the participant's perspective before a trial of the self-generated condition, with the green cylinder indicating the teleportation destination located in front of the subsequent door. **(C)** Virtual keypad with shuffled numbers where the door code has to be entered. The valid combination is displayed in black letters above and below the number pad. The black beam indicates the pointing direction. **(D)** View of the corridor before a trial of the externally generated condition, with the robot sphere floating in front of the door. **(E)** Keypad during the robot trial. Participants activate the robot with the controller, the keypad is locked, and tones are generated by the simulated robot only.

Participants sat comfortably inside an electrically shielded and acoustically attenuated chamber (IAC Acoustics, Niederkrüchten, Germany) while performing the experimental task. The technical setup is schematically depicted in Figure 1. The setup parts inside the chamber are displayed by photograph on Figure 2A. For immersion into VR, participants wore an HTC Vive Pro Eye headset (HTC Corporation, Taoyuan City, Taiwan), featuring dual OLED displays with a combined resolution of 2,880 x 1,600 pixels, a screen refresh rate of 90 Hz, and a maximum field of view of 110°. Computations were performed on a Bestware XMG NEO 17 laptop (AMD Ryzen 9 5900HX CPU, 32 GB RAM, Nvidia RTX 3080 Mobile GPU). The built-in headphones of the HTC Vive were replaced with a Sennheiser HD 25-1 (70Ω) headset. For interactions with the VR, one of the HTC Vive's bluetooth hand-held controllers was used. To minimize input delay, a copper wire coil was added to the surface of the controller's touchpad, and the end of another thin copper cable was attached to the participants' thumb, using two small strips of adhesive tape. On contact, a signal was elicited, amplified and then fed to an Arduino UNO for processing. This allowed for near-instant detection of haptic contacts, with an accuracy better than one-tenth of a millisecond.

To achieve latency control between controller input and sound output, the Arduino would send a trigger to the PS/2 port of a Linux-based PC upon detecting a haptic contact at the copper wire coil attached to the controller. The Linux PC handled the script for latency manipulation and generated the auditory stimuli, played binaurally through the Sennheiser headphones. The minimum latency delay that was achievable with this processing chain was 10 ms, thanks to the sub-millisecond detection of haptic contacts circumventing the usual response time of the controller itself. The auditory stimulus was a 1,000-Hz sine tone of 50 ms duration presented at 70 dB(A). The tones' A-weighted sound pressure level was measured using binaural microphones and a sound level meter (Brüel and Kjær, Nærum, Denmark). Temporal synchronization between the auditory stimuli and the EEG triggers was achieved by connecting the EEG system (see below) with the parallel port of the Linux PC. This allowed for the transmission of precisely timed 8-bit format triggers to differentiate between the experimental conditions in the EEG data.

The Linux PC was operated on GNU/Arch Linux 4.5.1 containing the real-time kernel. Auditory stimulus generation and timing were performed in MATLAB R2015b using the Psychophysics Toolbox extensions (Brainard, 1997; Pelli, 1997). The 3D assets for the VR were modeled in Blender (2.93) and the final environment was implemented and operated in Unity (2019.4.28f1), using the SteamVR plugin (2.7.3) to handle the tracking of the VR devices and to access controller inputs from within Unity. The VR space was set up in Windows 11 running SteamVR (1.20.4).

#### 2.2.2. EEG recordings

EEG data were continuously recorded from a total of 32 active AgCl electrodes using a stationary actiCHamp EEG system (BrainProducts GmbH, Gilching, Germany). Twenty-seven electrodes were placed in an elastic cap (actiCAP SNAP) and positioned according to the international 10–20 system (Chatrian et al., 1985). Five additional electrodes were placed at the left and right mastoids, on the tip of the nose (serving as online reference) and below the left and right eye, but lower than the standard locations IO1 and IO2 to accommodate the VR headset (see Figure 2A). The ground electrode was placed at Fpz. Impedances were kept below 20 kΩ. EEG signals were amplified by the actiCHamp EEG system, recorded with the BrainVision Recorder software (Brain Products GmbH), and sampled at 500 Hz with a 249 Hz online lowpass filter to avoid aliasing.

To ensure compatibility with the head-mounted VR setup, electrodes could not be as evenly distributed over the head as usual in EEG experiments. In detail, the electrodes O1 and O2 could not be used due to their position interfering with the back-strap of the VR headset, and LO1 and LO2 would have collided with the display enclosure of the HTC Vive. Instead of O1, O2, LO1 and LO2, additional measurements were taken from FCz, CPz, C1, and C2. This denser spatial sampling at frontocentral electrodes was chosen because it reflects the expected topography of auditory ERP components (Alain and Winkler, 2012). The resulting 27 scalp electrode locations were Fp1, Fp2, F7, F3, Fz, F4, F8, FC5, FC1, FCz, FC2, FC6, T7, C3, C1, Cz, C2, C4, T8, CP5, CP1, CPz, CP2, CP6, P7, Pz, and P8.

### 2.3. Procedure

The experiment consisted of 12 blocks in which participants' task was to open doors by operating a virtual numeric keypad. In 10 of the 12 blocks, virtual keypresses generated auditory stimuli with different input delays (10 ms/50 ms/100 ms/150 ms/300 ms, two blocks per condition). The remaining two blocks served as motor-control condition, in which participants performed the same task but without auditory stimuli. Prior to the actual experiment and the preparation of the EEG recordings, participants underwent a VR-only training, in which they were familiarized with the controls and the task, and in which VR-inexperienced participants were able to test their tolerance to VR. The training phase consistently used the shortest latency condition (10 ms), and participants completed an entire block of trials. The scenarios presented in both the training and main experiments were identical. Specifically, participants used seated VR to navigate through an approx. 2 m wide corridor divided into 4.3 m long sections by locked doors. The participants' task was to teleport to the next section of the corridor by directing the HTC Vive controller held in their right hand toward the designated teleportation marker located in front of the subsequent door, followed by pressing the trigger button on the controller with their index finger (Figure 2B).

Located at each door was a virtual numeric keypad that required the entry of a four-digit combination to open. The valid access code was constantly displayed above the numeric keypad, while the input was made by pointing a black beam emanating from the controller at a digit on the number pad, followed by confirming the input through a light tap of the touchpad on the controller using the participant's thumb, without pressing the touchpad down (Figure 2C). Each digit entry (except in the two motor-control blocks) was followed by a brief sound occurring 10 ms, 50 ms, 100 ms, 150 ms, or 300 ms after activation of the touchpad depending on the delay condition. Each touchpad activation and the following sound represented one trial to which event-related brain potentials were analyzed. To ensure a distinct temporal separation of the ERPs, the speed with which the digits could be entered was deliberately slowed down. First, the numeric keypad was made larger than usual (55 cm x 85 cm), thereby prolonging the time needed for moving the beam to the next digit's position. Second, the arrangement of the numbers on the keypad was randomized for each door, thereby prolonging the time needed for finding the next digit's position. Finally, input was blocked between consecutive digit entries for about one second, with the pointing beam turning off 500 ms after an input (to avoid potential visual effects during the targeted auditory ERP components)—and turning on again after another 500 ms, each with a jitter of +/-100 ms to prevent predictability. No visual feedback was provided to the participants. The door would open only if the four digits were entered correctly in sequence. If there was an incorrect entry, the display would show 'FALSE!' after the fourth input, and the entry process had to be restarted.

The blocks with sounds included a replay condition to record ERPs elicited by sounds without a motor act of the participant (i.e., externally generated). After every three doors, the door code input was taken over by a hovering robotic sphere that would flawlessly enter the code and generate a four-tone sequence (Figures 2D, E). The tone sequence was randomly selected without replacement from the pool of self-generated tone sequences that had been created by successful door openings of the participant in that block.

In each block, 15 doors had to be opened by the participant. In blocks with self-generated sounds, an additional 5 doors were opened by the robot sphere (externally generated sounds). Within a block, the delay of auditory stimuli remained constant. After each block with sounds, participants were asked whether they had noticed a delay between their button presses and the resulting sounds, and their verbal answers were taken down. The question did not enforce a strict choice between “yes” and “no,” but also allowed for answers such as “unsure,” “slightly,” or “in some trials.”

Of the 12 blocks in the experiment, the first five blocks included all five motor-auditory latency conditions exactly once, with the order randomly selected for each participant. The sixth block was always the condition without auditory stimuli (motor control). In blocks 7–12, the order of the first six blocks was reversed.

The whole experimental session lasted approximately 3 h to 3.5 h, including instructions and VR training, electrode application, the main task (1 to 1.5 h), electrode removal as well as ample breaks for the participants to maintain motivation and prevent fatigue.

### 2.4. Data analysis

#### 2.4.1. Participant judgments

Participants' judgments of system latency were categorized as 0 (no delay noticed), 2 (clear delay noticed), or 1 (answers falling in between the two extremes, including the participant being unsure, reporting a slight or minimal delay, or noticing delay on some trials but not others). The frequencies of the three categories were calculated separately per latency level.

#### 2.4.2. EEG data pre-processing

The EEG data were analyzed offline using MATLAB R2020b (The MathWorks Inc., Natick, USA) and the toolbox EEGLAB (Delorme and Makeig, 2004) version 2022.1. EEG data recorded below the left and right eyes were excluded from all analyses, as the placement of the electrodes (lower than the standard locations IO1 and IO2 to accommodate the VR headset) could not be maintained at a pre-defined anatomical position for all participants. In fact, these electrodes' signal had no added value for artifact rejection (eye-movement-related artifacts were hardly picked up there). For the removal of stereotypical artifacts, EEG data were decomposed into independent components (ICs) with the extended Infomax algorithm (Bell and Sejnowski, 1995). Prior to and only for the purpose of independent component analysis (ICA), a copy of the raw data was high-pass filtered using a Kaiser-windowed sinc finite impulse response (FIR) filter with a 1 Hz cutoff (Kaiser β = 5.65326, filter order: 9,056, transition bandwidth: 0.2 Hz, maximal passband ripple: −60 dB), the data were epoched into 1-s segments (not representing the actual experimental trials), and non-stereotypical artifacts were excluded based on joint probability and kurtosis with a threshold of three standard deviations. To identify ICs representing artifacts caused by eye movements, blinks, and muscle activity, the components were pre-labeled using EEGLAB's IClabel function (Pion-Tonachini et al., 2019) and then inspected visually. Components classified as eye or muscle activity with a certainty of more than 90% were marked for rejection and subsequently removed from the untreated EEG dataset (i.e., without the 1-Hz filter). The number of removed components per participant ranged from 2 to 10 (Mean = 7.0, SD = 2.2). The ICA-corrected EEG data were filtered using a windowed-sinc high-pass FIR filter with a 0.1 Hz cutoff (Kaiser β = 5.65326, filter order 9,056, transition bandwidth: 0.2 Hz, maximal passband ripple: −60 dB), and a windowed-sinc low-pass FIR filter with a 45 Hz cutoff (Kaiser β = 5.65326, filter order 184, transition bandwidth: 10 Hz, maximal passband ripple: −60 dB).

The resulting data were segmented into epochs of 600 ms duration for all conditions with sounds (separated into externally generated and self-generated with the five different delay levels). Epochs ranged from −100 ms to 500 ms relative to the onset of the auditory stimulus (Pinheiro et al., 2019). They were baseline-corrected using the interval from−100 ms to 0 ms relative to sound onset. In the motor-control condition (i.e., without sounds), epochs of 890 ms duration were extracted from −90 ms to 800 ms relative to the tap on the touchpad. The longer epoch duration was chosen to compensate for the different auditory delays for later alignment when subtracting the motor-only ERPs from the self-generated auditory ERPs (Elijah et al., 2016). The start of the motor-control epochs (−90 ms) matched the start of the epoch in the 10-ms-sound-delay condition, and the end of the motor-control epochs (800 ms) matched the end of the epoch in the 300-ms-sound-delay condition. The motor-control epochs were baseline-corrected separately for each subtraction, using the interval from −100 ms to 0 ms relative to when the sound would have occurred in the respective comparison condition with self-generated sounds.

Epochs with amplitude changes exceeding 150 μV on any channel were rejected from further analysis. This left one participant with < 70% remaining epochs after artifact rejection; the corresponding dataset was excluded from further analysis (see above). The remaining datasets showed an average data loss of 3% across conditions and participants, with a maximum data loss per participant of 14% across conditions. The range of remaining epochs per participant and condition was 76 to 249 in the self-generated sound conditions (Mean = 147.8, SD = 22.9), 147 to 200 in the externally generated sound condition (Mean = 187.3, SD = 13.9), and 80 to 218 in the motor-control condition (Mean = 147.4, SD = 30.3).

It should be noted that the variability in the number of epochs between participants did not primarily stem from EEG artifacts (as indicated by the average data loss of only 3%), but from the requirement to repeat the task if not all four digits were correctly entered. As explained above, a copper wire coil was attached to the touchpad of the controller to enable faster detection of haptic input and precisely manipulate the timing of the sounds. However, due to the lower sensitivity of the controller's touchpad compared to the wire, participants occasionally triggered only the wire (not the touchpad) and thus failed to successfully input a number. Despite this, participants were still presented with sounds since this was controlled by the copper wire. As a consequence, they did not realize that the virtual keypad input was unsuccessful until after trying to enter the fourth digit. Trials with this type of error were classified as ‘false' and repeated. This happened more often than expected and consequently, a substantial number of additional (repeated) keypad inputs was performed overall. As participants could not have recognized the error while performing the task (since the sounds were presented and no other feedback indicated an unsuccessful number entry), these additional trials were not excluded from EEG analysis.

#### 2.4.3. Event-related brain potentials

Data from the remaining 23 participants were used to form single-subject and grand-average ERPs per condition. All epochs were averaged per electrode and condition within each participant, resulting in single-subject ERPs per condition. To allow for comparison of the ERPs elicited in the externally generated sound condition (without a motor act by the participant) with the ERPs elicited in the self-generated sound conditions (with a motor act by the participant), brain activity elicited by the motor act first had to be separated from brain activity elicited by processing the auditory stimuli. To subtract the motor component, the ERP elicited in the condition without sounds (motor control) was subtracted separately for each participant and electrode from each of the ERPs elicited in the conditions with self-generated sounds (i.e., five difference waves calculated as ERP[motor-corrected] = ERP[self-generated sound] minus ERP[motor-control]). The motor-control and self-generated sound ERPs were temporally aligned to the tap on the touchpad for the purpose of this subtraction (Elijah et al., 2016). The resulting difference wave, re-aligned to sound onset, reflects the motor-controlled processing of self-generated sounds. The grand-average ERP was then calculated by averaging ERPs across all participants for each electrode and each of the six conditions (externally generated, self-generated with 10/50/100/150/300 ms delay). Experimental conditions will be referred to as “10 ms,” “50 ms,” “100 ms,” “150 ms,” “300 ms” for self-generated sounds and “external” for the sounds replayed by the robot sphere.

**Figure 3:**
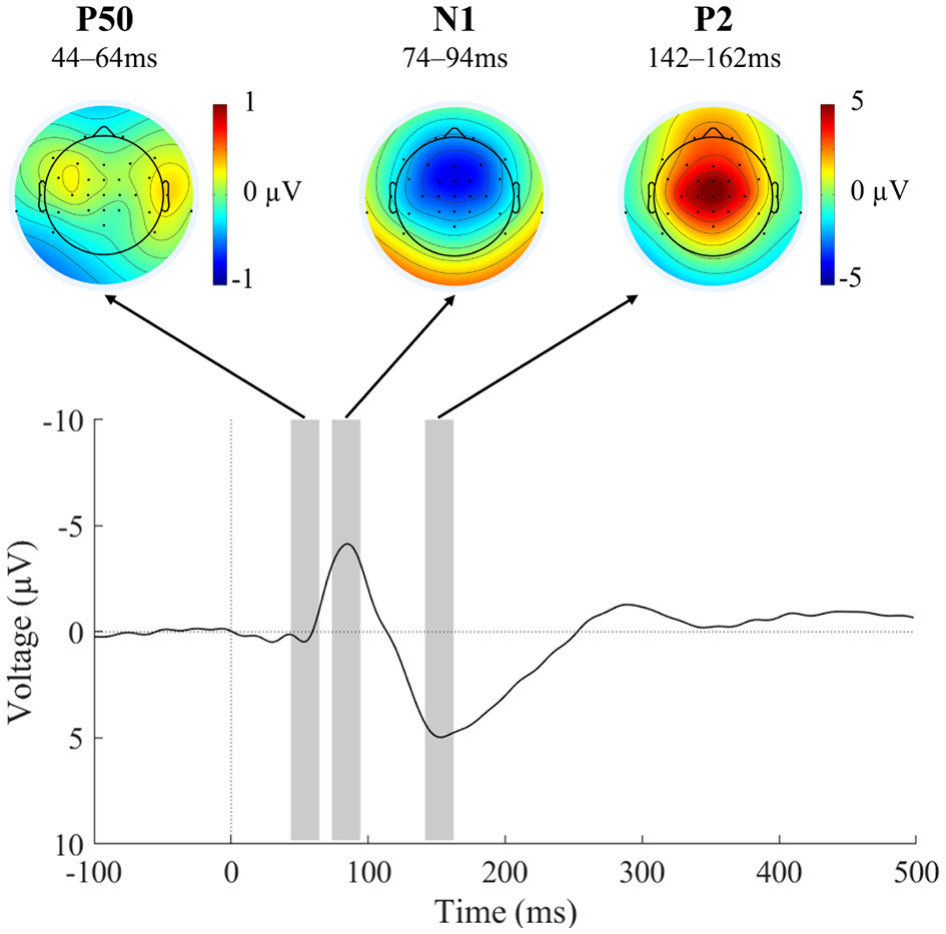
EEG results across conditions for component identification. Grand-average ERP (*N* = 23) at the frontocentral electrode cluster averaged across all motor-corrected delay conditions and the external condition, as well as scalp voltage distribution of the identified ERP components P50, N1, and P2. Negative ERP voltage is plotted upward. The average across all conditions was used for an unbiased identification of component windows. The resulting component windows used for topography plots and statistical testing are indicated by gray rectangles. Please note the different scale in the topography plot of the P50 component.

In order not to introduce any bias into the selection of the intervals for analyzing the auditory ERP components, the ERP traces were averaged across all six conditions that were to be compared later (Keil et al., 2014). This all-condition average showed the expected morphology of auditory ERP components (Figure 3), with elicitation of the P50, N1, and P2 components each peaking at frontocentral electrodes. A cluster of seven electrodes centered around FCz (Fz, FC1, FCz, FC2, C1, Cz, C2) was used to identify component latencies and quantify component amplitudes. Peak amplitudes were identified at 54 ms (P50), 84 ms (N1), and 152 ms (P2). For peak-window-based statistics, time windows of 20 ms were symmetrically chosen around the component peaks (Elijah et al., 2016) and cross-checked to adequately reflect the component morphology in the single-subject ERPs. Component amplitudes were thus measured from 44-64 ms (P50), 74-94 ms (N1), and 142-162 ms (P2). It should be noted that peak-picking procedures are being criticized for their circularity (“double dipping”) (Kriegeskorte et al., 2009; Luck and Gaspelin, 2017). In the current case, the research question is not whether the auditory ERP components of interest are *elicited*, but whether their amplitude is *modulated* by condition. Thus, choosing the latency ranges based on an average of all conditions does not introduce circularity into the analysis.

### 2.5. Statistical analysis

P50, N1, and P2 component amplitudes were quantified separately for each participant and condition in the pre-determined latency ranges. The analysis focuses on the N1 and P2 components as in most previous studies on suppression of self-generated sounds (SanMiguel et al., 2013b). Since it was apparent from the current waveforms that slow ERP drifts affected the P50 and N1 latency ranges alike, measuring baseline-to-peak N1 amplitudes would not provide an adequate assessment of the N1 component amplitude. N1 amplitudes were therefore measured as peak-to-peak amplitudes (N1 amplitude minus P50 amplitude) to cover the full amplitude of the N1 component. For P2 component amplitudes, baseline-to-peak measurements were used as there was no apparent overlap. N1 and P2 amplitudes were first compared between self-generated and externally generated sounds by two-tailed, paired-sample *t*-tests using the average of all five self-generated sound conditions. N1 and P2 amplitudes were then submitted to repeated-measures analyses of variance (rmANOVAs) with the five-level factor delay (“10 ms,” “50 ms,” “100 ms,” “150 ms,” “300 ms”) to assess the effect of the delay manipulation. Significant main effects in the rmANOVAs were followed up by linear trend tests to assess the effect trajectory across the delay values. In case of a significant violation of the sphericity assumption according to Mauchly's test, *p*-values were adjusted using the Greenhouse-Geisser correction and are reported alongside the uncorrected degrees of freedom and the Greenhouse-Geisser epsilon. Statistical analyses were conducted with IBM SPSS Statistics 29.0.0.0. The alpha level for all statistical tests was set to 5%. Following Mordkoff (2019), all effects are reported alongside with adjusted partial eta square as a measure of effect size.

Participants' judgments of system latency were statistically analyzed by comparing the frequency distribution of the three response categories (delay noticed / unsure / not noticed) across the five latency levels with a χ^2^ contingency test.

## 3. Results

As denoted above, the auditory P50, N1, and P2 components were elicited with the expected time-course and topographies (Figure 3; Alain and Winkler, 2012).

**Figure 4:**
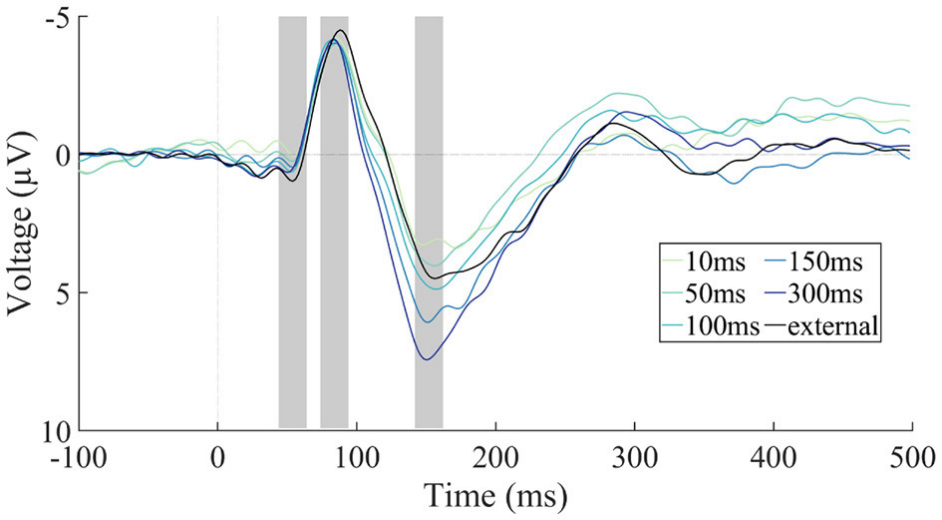
EEG results by condition. Grand-average (*N* = 23) ERP responses at the frontocentral electrode cluster separated by condition. The five self-generated conditions with different latency delay values are shown after being adjusted for the motor component, and contrasted with the externally generated condition (without motor correction). Negative voltage is plotted upward. The P50, N1, and P2 latency ranges are marked with gray rectangles.

**Figure 5:**
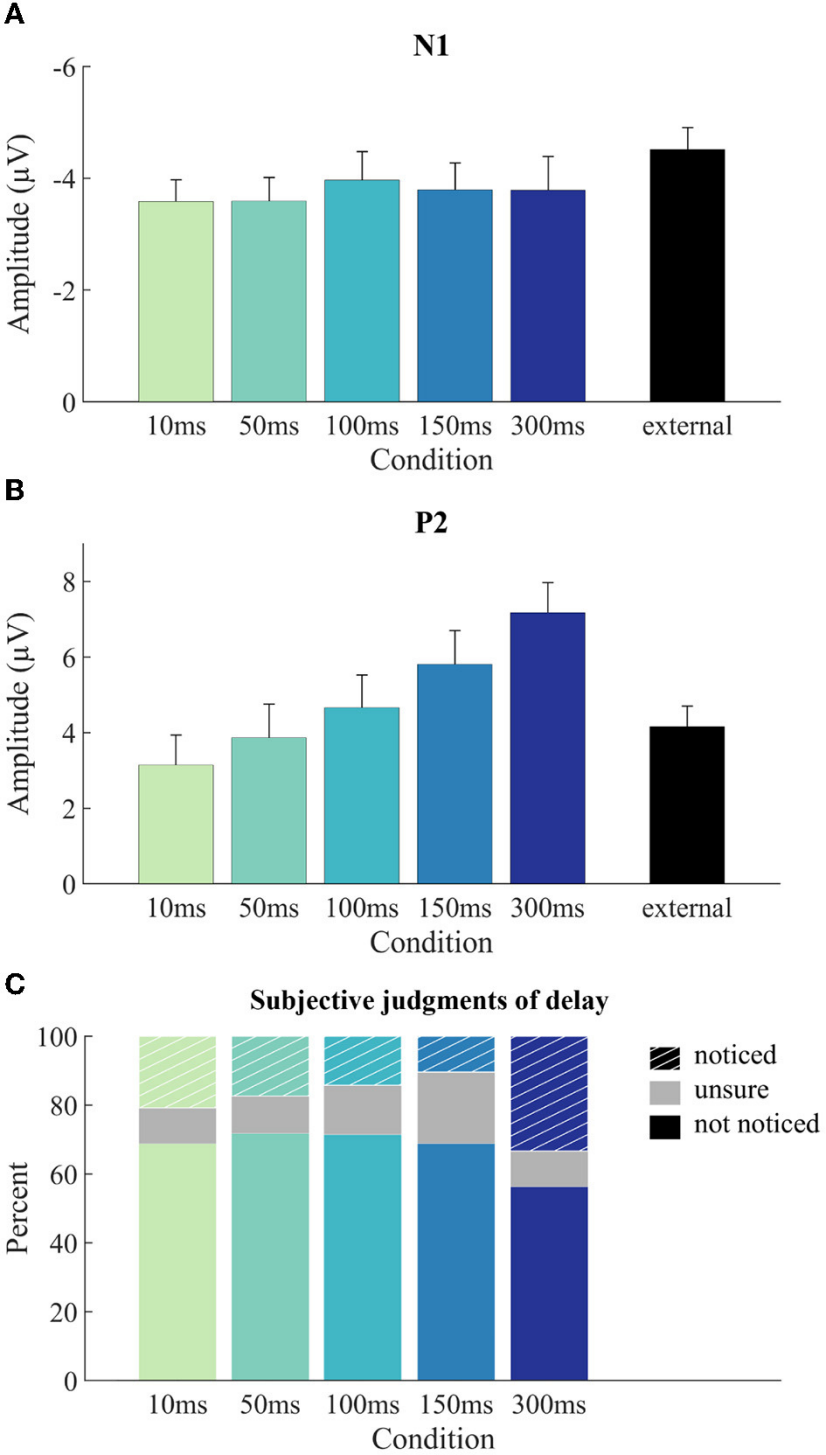
ERP component amplitudes and subjective judgments by condition. Mean component amplitudes for N1 **(A)** and P2 **(B)** per condition together with between-subjects standard error of the mean. N1 amplitudes **(A)** are determined as peak-to-peak values (N1 minus P50), P2 amplitudes **(B)** are determined as baseline-to-peak values. **(C)** Frequency distribution of the three subjective-judgment categories for noticing the delay across the five latency delay levels.

ERPs were modulated in different time-ranges by the sounds being self- vs. externally generated and by the delay of self-generated sound presentation (Figure 4). The amplitude of the N1 component was significantly larger for the external condition than for the mean of the five self-generated conditions, *t*(22) = −3.54, *p* = 0.002, adj. η_p_^2^ = 0.333, indicating N1 suppression for self-generated sounds (Figure 5A). The comparison of the five self-generated conditions with an rmANOVA revealed no significant main effect for the factor delay, *F*(4,88) = 0.30, *p* = 0.879, adj. η_p_^2^ = −0.032, indicating that N1 suppression was not modulated by delay.

The amplitude of the P2 component did not significantly differ between the external condition and the mean of the five self-generated conditions, *t*(22) = −1.65, *p* = 0.112, adj. η_p_^2^ = 0.070, indicating no overall P2 suppression for self-generated sounds (Figure 5B). P2 amplitude for self-generated sounds was, however, modulated by the delay of sound presentation, with longer delays corresponding to larger P2 amplitudes (Figure 5B). This was confirmed by a significant main effect of delay in the rmANOVA, *F*(4,88) = 10.48, *p* < 0.001, adj. η_p_^2^ = 0.292, ε_*GG*_ = 0.61. A follow-up linear trend test confirmed that the increase in P2 amplitude was systematic to the increase in delay, *F*(1,22) = 21.37, *p* < 0.001, adj. η_p_^2^ = 0.470.

Since the P2 amplitude of the external condition unexpectedly fell in between the P2 amplitudes of the self-generated conditions, and since the latter were significantly modulated by delay, we conducted *post-hoc* two-tailed paired-sample *t*-tests comparing each delay condition individually with the external condition. These comparisons yielded significant differences for “150 ms” [*t*(22) = −2.52, *p* = 0.020, adj. η_p_^2^ = 0.188] and “300 ms” [*t*(22) = −4.73, *p* < 0.001, adj. η_p_^2^ = 0.482], but not for “10 ms” [*t*(22) = 1.75, *p* = 0.094, adj. η_p_^2^ = 0.082], “50 ms” [*t*(22) = 0.42, *p* = 0.681, adj. η_p_^2^ = −0.037] and “100 ms” [*t*(22) = −0.78, *p* = 0.442, adj. η_p_^2^ = −0.017]. We note that only the *p*-value of the “300 ms” condition is significant against a Bonferroni-corrected alpha level of 0.01.

Overall, these results indicate a modulation of N1 (but not P2) by self-generation, and a modulation of P2 (but not N1) by delay between the motor act and the sound.

Participants' judgments of having noticed a delay were quite evenly distributed across the latency levels (Figure 5C). Numerically, there were slightly more reports of having noticed a delay for the 300 ms latency level, but this pattern was not significant as determined by the χ^2^ contingency test, χ^2^(8) = 11.69, *p* = 0.116.

## 4. Discussion

The current study was designed to evaluate whether brain activity elicited by user-generated sounds can be used to assess system latency in VR. To this end, we implemented different values of motor-auditory delay in a VR task in which users generated sounds *en passant* while operating a virtual keypad. Our EEG results show a graded modulation of the P2 ERP component by system latency, opening up the possibility of using P2 amplitude for detecting inadequate latencies in future studies. The N1 component was not modulated by system latency, but by the origin of the sound, replicating the established sensory suppression effect of self-generated relative to externally generated sounds.

Prior studies in which participants pressed buttons to generate sounds (without a VR context) had yielded inconsistent results in terms of the ERP components that would be affected by latency delay manipulations. While some studies showed that N1 suppression decreases from 0 ms to 100 ms delay (Elijah et al., 2016; Oestreich et al., 2016), in other studies the amount of N1 suppression was not affected by delay up to 100 ms (Pinheiro et al., 2019) or even up to 200 ms (Timm et al., 2016). These different N1 outcomes may partly be due to design differences. For instance, Pinheiro et al. (2019) included delayed and non-delayed sounds within the same blocks, thereby mixing effects of delay with effects of expectation violations across trials (speaking in terms of system latency, manipulating latency jitter in addition to latency delay). On the other hand, Elijah et al. (2016) as well as Timm et al. (2016) were specifically interested in having participants adapt to a certain delay and thus used prolonged presentations with the same delay. In the current study, participants experienced about 75 trials per delay value before moving on to another delay value (with the precise number of trials depending on how many times they had to re-enter a code because the door would not open). Based on the trial numbers employed in the non-adapted conditions by Elijah et al. (2016), it seems unlikely that our participants adapted to the delay value of each block of the current study to the extent of not showing N1 modulation by delay anymore.

Not only in terms of trial number, but also in terms of sample size, the current study was well in the range of prior lab studies with similar delay manipulations (Klaffehn et al., 2019; Pinheiro et al., 2019; also Elijah et al., 2016, taking the between-subject manipulation into account) and rather at the upper end of comparable VR-EEG studies (see review by Putze et al., 2022). In terms of EEG data quality, although 64-channel EEG would have been advantageous, ICA based on 32 channels as applied here already leads to a substantial improvement in signal-to-noise ratio (Klug and Gramann, 2021). It thus seems unlikely that the absence of an effect of delay on N1 amplitude was due to noise or lack of statistical power. Rather, we conclude that N1 is not (or not sufficiently) sensitive to latency delay in the current setting, and thus does not lend itself toward a read-out of latency adequacy from users' brain activity.

N1 was, however, affected by whether a sound was self- or externally generated, thereby replicating the well-established N1 suppression effect (Horváth, 2015) and confirming the validity of our external control condition. Having the robot operate the virtual keypad is similar to “social” versions of the classical button press task in which there is another agent performing the same action (Weiss et al., 2011; Ghio et al., 2021). In the current study, participants could not see the robot's movements, and hence there was no temporal predictability based on action observation (though effects caused by observing event initiation by another agent should be small in any case, see Weiss and Schütz-Bosbach, 2012; Ghio et al., 2021; note that N1 suppression occurs even when predictability is strictly controlled for by means of visual input, see Klaffehn et al., 2019). The sound sequences generated by the robot's keypad operation constituted replay versions of the participant's self-generated sequences, ruling out physical differences between the self- and externally generated versions. Thus we infer that genuine motor-sensory attenuation led to N1 suppression in the current study.

The current P2 results were opposite to those of the N1 component: P2 did not show a response difference between self- and externally generated sounds, but it was clearly modulated by the latency delay of self-generated sounds. This pattern of results (N1 responding to self-generation, P2 responding to delay) is consistent with the studies by Timm et al. (2016) as well as Pinheiro et al. (2019). Further studies manipulating delay (Whitford et al., 2011; Elijah et al., 2016; Oestreich et al., 2016) did not analyze the P2 component, thus it is difficult to compare their results to the current ones in this respect. Klaffehn et al. (2019) contrasted much more extreme delay values (0 ms vs. 750 ms) and added visual cues to maintain temporal predictability in the long-delay condition. Their pattern of results is also consistent with the current one, with the P2 showing an effect of delay but not of self-generation (when predictability is controlled for).

It is worthy to note that the absence of a self-generation effect on the P2 becomes apparent only by examining all five self-generated conditions (Figure 4): Had we applied only the 10 ms and the 50 ms or 100 ms delay conditions, we might have concluded that there is a tendency toward P2 suppression for 10 ms delay but not anymore for 50 ms or 100 ms delay. Yet the ERP trajectories for 150 ms and 300 ms delay indicate that the P2 modulation is not related to bringing the amplitude “back up” to that of the externally generated condition, but clearly exceeds that level. This finding illustrates the benefit of a systematic examination of a broad range of delay values. The modulation of P2 amplitude beyond the externally generated condition is in contrast to our initial hypothesis that the amplitudes by externally generated sounds would constitute an upper bound for the observable self-generated sound amplitudes (as shown by Blakemore et al., 1999, for perceptual judgments). There are two possible interpretations of the pronounced ERP differences: First, there might be fundamental processing differences between the self-generated and externally generated sound conditions in the P2 latency range, which would imply that additional cognitive processes are at play and overlay the ERP comparison. One candidate process would be a variation in attentional state: During external sound generation, participants had no own task—the door-opening task was performed by the robot, and watching the robot perform this task might be less attentionally engaging than opening the door oneself. However, devoting more attention to the sounds should have led to *less* positive P2 amplitudes in the self-generated conditions due to an overlaid negative difference or processing negativity (Näätänen, 1982; Saupe et al., 2013), whereas we found *more* positive P2 amplitudes in the self-generated conditions with high delay relative to external sound generation. Moreover, our task deliberately used *en passant* presentation of sounds to reduce attentional differences between the self-generated and external conditions (as opposed to lab studies in which participants arguably wait for the self-generated sound to occur as this is part of their task template; see Bäß et al., 2008).

Hence we consider a second interpretation more likely: If we take P2 amplitude as a measure of how difficult it is for the perceptual system to integrate the sound into a sensorimotor reference frame of the current environment, it might actually be “worse” to experience a self-generated sound with considerable delay than it is to experience an externally generated sound. This interpretation is in line with a previous finding of reduced P2 for self-generated sounds when the perceived control over sound generation is high (Seidel et al., 2021). The authors suggest that while early processing (in the N1 range) is affected by predictive action-related sensory suppression, later processing (in the P2 range) is rather impacted by a postdictive judgment of agency. Timm et al. (2016) similarly argue that—relative to N1—the P2 component might be related more directly to perceived agency. Thus, the detrimental consequences of high delay values (specifically, 150 ms and 300 ms relative to the button press) might reflect a more effortful process of forming agency judgments. This would be consistent with system design guidelines classifying such amounts of delay as beyond the acceptable range (Attig et al., 2017). The delayed occurrence of the sound might be particularly problematic in a context where the user cannot verify by other means whether their motor input was received by the system or not. We deliberately refrained from providing additional immediate feedback that the touchpad was pressed, for instance via a visual confirmation signal, because then delaying the sound would involve not only a delay between button press and sound but also a mismatch between the two sensory events, which would confound the ERPs. However, delayed sound occurrence might have added insecurity for our participants regarding the success of their action, thereby posing challenges to smooth operation of the virtual keypad. This difficulty might have extended to the motor-only control condition as well, in which participants did not receive any feedback as to whether the touchpad was truly pressed or not due to the absence of auditory stimuli. However, due to the block-wise administration of the motor-control condition, participants knew that no sound was to be expected, and such knowledge might be easier to adapt to than a changing delay from block to block. In future studies we will probe the boundaries of such adaptation effects by contrasting block-wise with trial-wise manipulations of sound delay (including “infinite” delay as a motor-only control) and examining the resulting ERP effects.

The robust modulation of P2 amplitude by delay in the current studies is promising in terms of using this as a read-out for detecting inadequate system latencies from users' brain activity. The work of Gehrke et al. (2019, 2022) has already demonstrated that this is possible for a different type of ERP modulation: They introduced temporal mismatches between sensory stimuli received by the visual and haptic modalities, and showed that these mismatches are accompanied by prediction-error-related ERP components (Gehrke et al., 2019). In a follow-up study, they showed that this ERP effect can actually be used as input for a classifier that detects trials with visuo-haptic mismatches solely based on participants' EEG data (Gehrke et al., 2022). Their ERP-based classification reached an accuracy of 77%, whereas classification based on behavioral data barely exceeded chance level (55%). Similarly, Alsuradi et al. (2021) showed that delayed haptic feedback (relative to a visual collision) yields notable EEG changes including enhanced oscillatory power in the theta band as well as amplitude enhancements in the P2 latency range. This illustrates the enormous potential of using EEG for unobtrusive continuous evaluation of VR environments (Wang and Suh, 2021).

Other studies have pursued machine-learning approaches similar to Gehrke et al. (2022), with ERP correlates of movement error attribution to oneself or another VR agent yielding a single-trial classification accuracy of 73% (Dimova-Edeleva et al., 2022), and ERP correlates of visuospatial tracking errors reaching a single-trial classification accuracy of 85% (Si-Mohammed et al., 2020). In the study by Si-Mohammed et al. (2020), two other types of VR errors (erroneous feedback and visual anomalies in the background) could not reliably be detected at the level of the users' EEG. This illustrates that EEG is a promising tool for unobtrusive VR evaluation, but gaining information from it requires thorough consideration of the specific EEG features that can be read out, and of the tasks or events in which those EEG features are robustly elicited. Si-Mohammed et al. (2020) emphasize that the VR events associated with the targeted system anomaly should be part of an ecologically valid task set for the user (see also Yazmir and Reiner, 2022). This is in stark contrast to previous approaches in which (auditory) ERPs in VR were elicited by task-irrelevant probe stimuli to gain a measure of the users' spare perceptual or cognitive capacity, allowing for indirect inference from that measure on the users' presence in VR, and then again indirect inference from the users' presence on the adequacy of VR parameters (e.g., Kober and Neuper, 2012; Burns and Fairclough, 2015).

Measuring direct correlates of VR errors or anomalies (such as latency delay in the current study) allows for more specific inference, but also poses stronger requirements on how to seamlessly integrate appropriate test events into the VR. In other words, not only should the measurement technique be unobtrusive (as is the case for any physiological or behavioral measure that does not take the user out of the VR context for the sake of measuring), but also the event presentation itself. Unobtrusive event presentation means that the events used for VR quality evaluation are a natural element of the task users are performing in VR (as opposed to presenting additional stimuli just for the sake of VR evaluation). The incidental nature of sound presentation as a byproduct of virtual keypad operation and door opening is also what distinguishes the current work from many previous lab studies on self-generation of sounds (Bäß et al., 2008; Whitford et al., 2011; SanMiguel et al., 2013b; Elijah et al., 2016; Oestreich et al., 2016; Pinheiro et al., 2019), where participants might be waiting for the sensory consequences of their actions as they have no other task besides pressing buttons and listening to the resulting tones.

The transition from the “button-press-for-tone task” (Elijah et al., 2016) to the incidental presentation of sounds in an engaging VR task came with the additional challenge of manipulating motor-auditory latencies in VR down to values smaller than what is feasible with current VR hardware and software. In basic lab paradigms in which button presses lead to sound presentation, it is possible to ensure near-immediate presentation of sounds by an appropriate combination of hardware (button, soundcard) and software (response poll, sound initiation) elements—though the labels “0 ms” or “immediate” might be somewhat optimistic for the non-delayed condition even in many of those settings. In the current setting, 10 ms delay was the absolute minimum that was achievable with our custom-built hardware shortcutting the software-based VR latency delays. One concern might be that participants accustomed to modern VR controllers would perceive a delay of 10 ms as unrealistically fast and thus experience an illusory lack of agency over sound generation. Such impression would, however, not have affected N1 suppression (Timm et al., 2016; Seidel et al., 2021), and would have affected P2 in the opposite direction of what we observed. It is thus unlikely that such illusory lack of agency affected the current results. On the other end, it might be questioned whether participants were accustomed to longer delays based on their personal VR experience and thus more quickly adapted to the blocks with 50, 100, and 150 ms delay than they would have in comparable button-press paradigms (Elijah et al., 2016). However, such higher-order effects of participants' expectations regarding hard- and software response latencies would typically not extend to the 300 ms condition, and this condition was not notably different from all others in terms of ERPs. Thus altogether, we consider it unlikely that participants' expectations regarding VR controller latencies shaped the ERP responses to a great extent.

In terms of participant judgments, we found surprisingly little influence of the actual delay value on the subjective reports of noticing a delay. Even the 300 ms latency level was not identified as clearly delayed by the majority of participants. A possible explanation is that the question was asked at the end of each block, where adaptation to the delay value of the just-experienced block would be maximal (whereas the ERPs were aggregated across the whole block, leaving more room for finding differences by delay even if adaptation took place throughout the block). Alternatively, the results might indicate that ERPs are sensitive even before users are clearly aware of poor system latency in some cases, as has been observed with user performance data as well (Martens et al., 2018).

The current work as well as the aforementioned studies showing EEG/ERP correlates of other types of VR interface errors (Gehrke et al., 2019, 2022; Si-Mohammed et al., 2020; Alsuradi et al., 2021; Dimova-Edeleva et al., 2022; Yazmir and Reiner, 2022) all have in common that they are examining brain activity elicited by physically different events (i.e., immediate vs. delayed sound presentation, matched vs. mismatched timing of multisensory information, correct vs. erroneous VR response to user input). This is promising for identifying system glitches in contexts in which the VR itself cannot be as precisely controlled as in these research settings. It would be even more beneficial if this could be brought to the next level, using EEG/ERPs not only for distinguishing between physically different system responses but between individual user judgments of the same system response. For example, the same latency delay might be perceived as acceptable by some users whilst other users might notice the delay and perceive it as distracting. Identifying which part of the ERP codes for such individual (and implicit) judgments of adequacy, would require a combination of EEG measurements with user judgments – taking the downside of interfering with the process being measured for the sake of obtaining “ground truth” on the users' evaluation of the experienced VR latencies. Obtaining different ERP trajectories for diverging subjective evaluations of the same objective latency delay would also be highly insightful for basic research on self-generation effects, which is still struggling with potential alternative explanations based on temporal proximity of the motor act and the sound (see review by Horváth, 2015). Ruling out this physical confound is notoriously difficult (Klaffehn et al., 2019), yet a promising starting point might be to compare users that are accustomed to different latency delays based on their VR experience and on the type of VR input devices they are accustomed to. If successful, this approach would benefit theoretical models of sensorimotor prediction and practical applications for unobtrusive VR quality evaluation alike, thereby truly bridging basic, translational and applied research in cognitive neuroergonomics (Gramann et al., 2021).

## Data availability statement

The pre-processed EEG data (single-subject averages) supporting the conclusions of this article are available alongside with the analysis code in a publicly accessible repository: https://osf.io/kb9vf/. The raw EEG data will be made available by the authors upon request, without undue reservation.

## Ethics statement

All study procedures were reviewed and approved by the applicable local ethics review board (Ethikkommission HSW, Chemnitz University of Technology, Case no. V-346-PHSFKS-Latenz-21072019). The participants provided their written informed consent to participate in this study.

## Author contributions

AB and JK acquired funding. AB, JM, JK, and SF contributed to conception and design of the study. JM and SF prepared the VR model as well as the custom-built hardware for stimulus presentation and response acquisition. JM, SF, and SG collected the data. JM and SF analyzed the data. AB and SG reviewed the data analysis. SF and AB wrote the first draft of the manuscript. All authors contributed to manuscript revision, read, and approved the submitted version.
